# A rare case of pancreatitis caused by pancreas divisum in a middle-aged patient and treated with stent implantation: Case report

**DOI:** 10.1097/MD.0000000000048038

**Published:** 2026-03-13

**Authors:** Nagham Alsaid Abdullah Altwer, Bayan Alsaid

**Affiliations:** a Faculty of Medicine, Damascus University, Damascus, Syria; b Laboratory of Anatomy, Faculty of medicine, Damascus University, Damascus, Syria.

**Keywords:** congenital abnormalities, pancreas divisum, pancreatitis, stent migration

## Abstract

**Rationale:**

Pancreas divisum is a congenital anatomical anomaly resulting from failure of fusion of the Santorini and Wirsung ducts during fetal development, with a prevalence of 5% to 14% in the general population. Although usually asymptomatic, it is associated with chronic and recurrent pancreatitis.

**Patient concerns:**

A 47-year-old female presented with severe abdominal pain, diarrhea, vomiting, and signs of dehydration for several days, with a delayed diagnosis.

**Diagnoses:**

Magnetic resonance cholangiopancreatography showed the main pancreatic duct with a separate estuary over the collecting duct. The accessory pancreatic duct had a common outlet with the collecting duct.

**Interventions:**

Endoscopic retrograde cholangiopancreatography with minor papilla papillotomy and double plastic stent implantation was performed.

**Outcomes:**

During the 1-year follow-up, the patient remained well without recurrence of pancreatitis.

**Lessons:**

Pancreatitis due to congenital abnormalities is infrequent but should be considered when common causes are not identified.

## 1. Introduction

Pancreas divisum is a congenital anatomic anomaly that occurs when the ventral and dorsal portions of the pancreas fail to fuse during the 8th week of fetal development.^[1]^ Pancreas divisum may cause recurrent pancreatitis crises, although most cases are asymptomatic.^[2]^

In around one-third of pancreatitis cases, the reason is difficult to detect from the initial study, as neither gallstones nor a history of alcohol consumption can be identified. Consequently, the condition is classified as idiopathic pancreatitis in such instances.^[3]^

Accessory sphincter stenosis is associated with a range of symptoms, including upper abdominal pain, nausea, vomiting, and it has the potential to lead to an intermittent pancreatitis.^[4]^

For diagnosis, magnetic resonance cholangiopancreatography (MRCP) is considered as a noninvasive method for imaging the pancreatic ducts. Its sensitivity reaches 52% and its specificity reaches 97%.^[5]^

Conservative management, such as antiinfection and antimedications, and pancreatic enzyme supplementation is applied as a 1st-line therapy. While, therapeutic intervention is performed for patient who suffer from recurrent pancreatitis crises or have a clear imaging change.^[6]^

Here we present a rare case of a middle-aged female patient suffering from pancreatitis due to pancreas divisum with delayed diagnosis due to the absence of clear radiological signs indicating the disease.

## 2. Case presentation

A 47-year-old female patient, with a history of hereditary hypercholesterolemia, did not complain of any previous digestive symptoms, she had no history of alcohol intake and no history of metabolic disease (including diabetes or dyslipidemia), she had no autoimmune conditions, no biliary lithiasis, and no previous surgical history, and she had a history with oral contraceptives for 15 days. She presented to the emergency department with a complaint of 2 days of watery diarrhea, vomiting of digested food, and severe pain in the right hypochondrium with pain spreading to the back (the cervical vertebrae) and right shoulder. The pain is not relieved by analgesics, wakes from sleep, and does not decrease in any position. Vital signs show a blood pressure of 100/70 mm Hg with signs of mild dehydration. The patient was diagnosed with gastroenteritis, and remained on normal saline serum for 2 hours and was then discharged.

**Figure 1.**
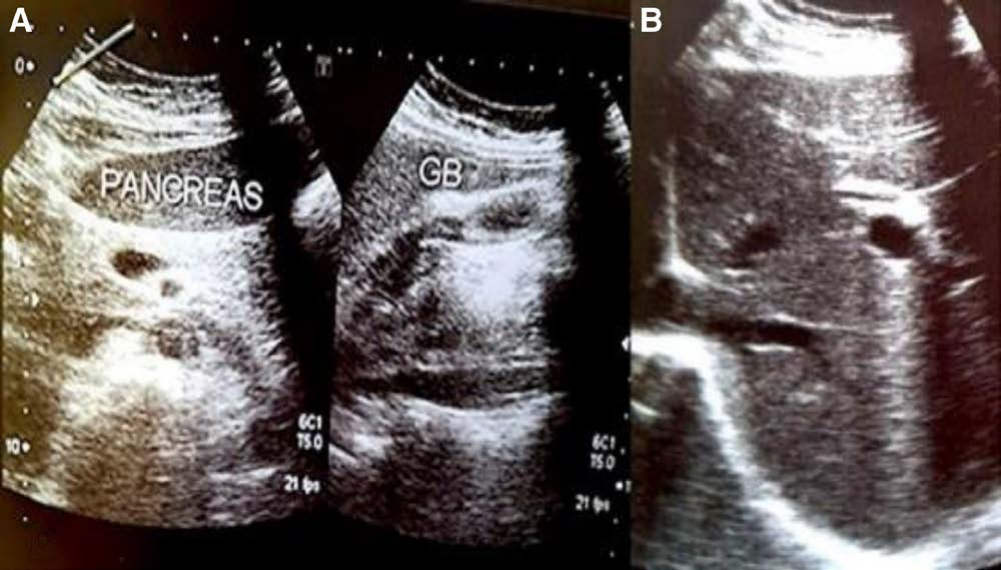
Abdominal ultrasound for a 47-year-old woman showed a normal pancreas, gallbladder (A) and liver (B).

The patient presented to the emergency department again 2 days later, complaining of persistent severe abdominal pain and persistent diarrhea with clear signs of dehydration. Laboratory tests showed an increase of white blood cells (neutrophils 75.5%, normal 40%–70%) (Lymphocytes 18.4%, normal 25%–40%). Abdominal ultrasound examination showed mild fatty infiltration around the cecum, appendix was not isolation and fluid in the peritoneal cavity around the right iliac region (Fig. 1), liver, spleen, pancreas, and gallbladder were normal (Fig. 1). The suggested diagnoses were gastroenteritis. The patient was then discharged after 2 hours.

**Figure 2.**
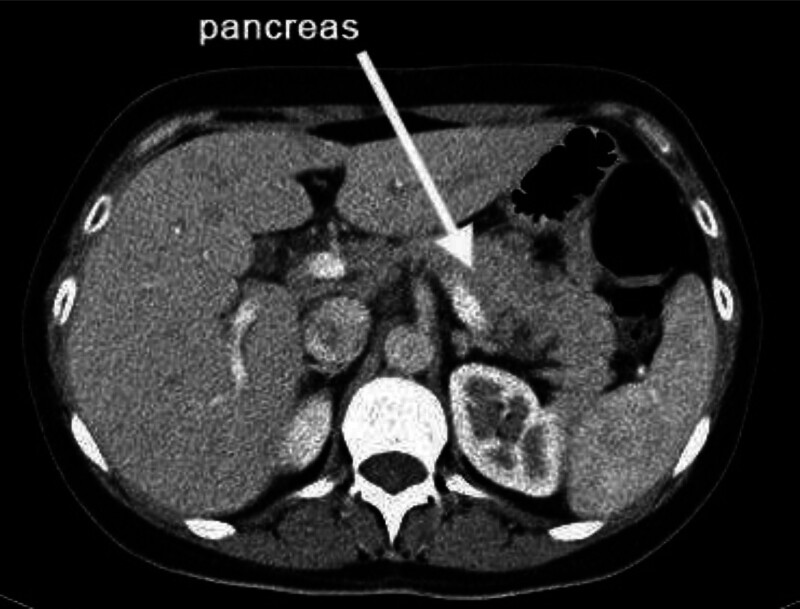
CT scan showed a normal pancreas (arrow). CT = computer tomography.

The patient represented to the emergency department 2 days later with severe pain in the right hypochondrium, inability to accept oral fluids, low vital signs, blood pressure 5/7 mm Hg, and clear signs of dehydration with the onset of fainting. Clinical examination showed signs of an acute abdomen. The patient was admitted to the hospital. Laboratory test results showed an increase of c-reactive protein (12.4 mg/L, normal 0–6 mg/L), an increase of liver enzymes serum glutamic oxaloacetic transaminase (64.4, normal <40), serum glutamic pyruvic transaminase (47, normal <45), and an increase of amylase (118 mg/dL, normal <100). An ultrasound examination was performed and showed a normal liver, pancreas, kidney, spleen, and a mild dilatation of the gallbladder. A computer tomography scan was performed, there were no dilatations of the main pancreatic ducts and there were no necrotic areas or pancreatic and peripancreatic fluid collections, liver and spleen were normal, there were a free fluid in the pelvic next to the right adnexa (Fig. 2). The patient was diagnosed with gastroenteritis and conservative treatment was applied. The patient remained on intravenous nutrition, intravenous antibiotics, and central analgesics. With continued follow-up, laboratory tests showed continued elevation of amylase values (450 U/L, normal <100) after 72 hours of conservative treatment, in addition to an elevation of lipase values 10 times the normal level (687 U/L, normal 10–55 U/L). Idiopathic pancreatitis was considered as diagnoses, and conservative treatment was continued with intravenous nutrition and no oral intake. During the time of admission, the patient was exposed to a hypoglycemic attack, continued pain, and needed central analgesics.

The patient remained stable with started to accept oral intake and was discharged after 15 days with continuing of central analgesia, and low-fat diet and took a pancreatic enzyme supplementation for 3 months. After discharging the patient continued follow-up to search for the reason factor of pancreatitis

**Figure 3.**
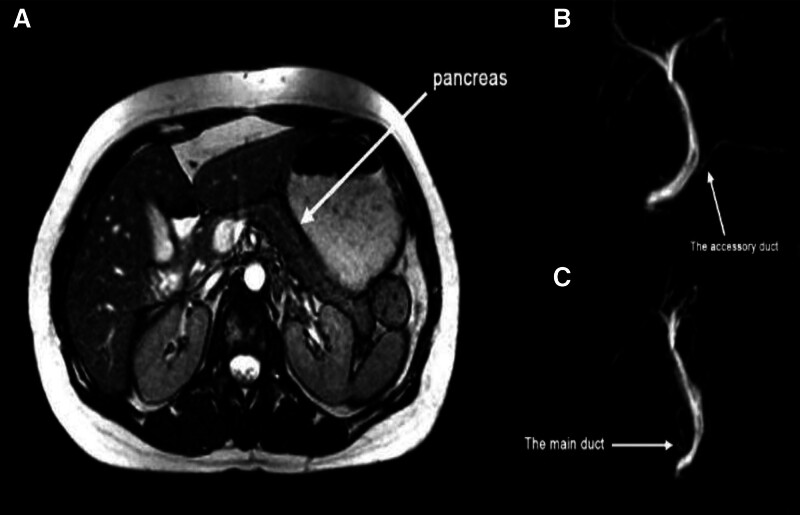
transverse section MRI (A) showed a normal pancreas (arrow), MRCP of pancreatic duct (B) showed the accessory duct have a common outlet with the collecting duct (arrow), MRCP of pancreatic duct (C) showed the main pancreatic duct have a separate outlet above the outlet of the collecting duct (arrow). MRCP = magnetic resonance cholangiopancreatography, MRI = magnetic resonance imaging.

During follow-up, the patient suffered from persistent symptoms of nausea, constipation, loss of appetite, intermittent abdominal pain of varying intensity, pain that eased after defecation and passing gas with bloating, weight loss, no fever, abdominal pain, and no visceral enlargement. Further investigation was performed. The results of the upper gastrointestinal endoscopy showed no signs of the disease except a 1.5 diaphragmatic hernia. A lower gastrointestinal endoscopy was performed and showed no signs of the disease except a colon spasm. Abdominal and pelvic ultrasound was normal, no dilatation of the bile ducts, homogeneous pancreas, and normal gallbladder after 4 months an MRCP was performed, in which the main pancreatic duct (duct of Wirsung) have a separate outlet above the outlet of the collecting duct, while the accessory pancreatic duct (duct of Santorini) have a common outlet with the collecting duct, the pancreas parenchyma appeared normal. The appearance is consistent with a pancreas divisum (Fig. 3). Two months later an endoscopic retrograde cholangiopancreatography (ERCP) was performed, in which the dorsal pancreatic duct was seen to be of normal dilatation with a narrowing at its beginning. An internal biopsy of the accessory papilla was performed and a 7 cm round stent was placed. After the procedure, the patient suffered from acute pancreatitis following the ERCP and was admitted to the hospital. She remained on conservative treatment and intravenous nutrition for 4 days. During follow-up, the patient was in a stable condition. After 2 months, the stent was spontaneously evacuated.

The patient remained stable after 1 year of follow-up, with a mild abdominal pain for unknown reason.

The primary etiological factors contributing to acute pancreatitis include gallstones and excessive alcohol consumption, the previous causes are considered the most common causes, in contrast, congenital anomalies are regarded as infrequent contributors, a divided pancreas is identified as the most frequent anatomical cause of pancreatitis.

## 3. Discussion

Pancreatitis represents a significant medical condition that can lead to detrimental health outcomes for affected individuals. Research indicates that the incidence of this disease rises at an approximate rate of 3%/yr from 1961 to 2016.^[7]^ The primary etiological factors contributing to acute pancreatitis include gallstones and excessive alcohol consumption, the previous causes are considered the most common causes, in contrast, congenital anomalies are regarded as infrequent contributors, a divided pancreas is identified as the most frequent anatomical cause of pancreatitis.^[8]^ In our case, we discussed one of the rare causes of pancreatitis, studies have shown that pancreatic divisum affects 15% of the population,^[6]^ but when using endoscopic retrograde cholangiopancreatography incidence reaches 25%.^[9]^ And for pancreatitis due to pancreatic divisum it is rarely to present in a middle-aged individual, whereas pancreatitis it is suspected in younger individuals or in the presence of a family history, Consequently, pancreatic divisum should not be neglected as one of the etiology of pancreatitis in elderly individuals or in those without a family history.^[10]^ As our case, it is not necessary to have a previous pancreatitis. Studies have shown that pancreas divisum is asymptomatic in 95% of patients.^[11]^ and it is often develops due to a secondary cause. According to the patient history, it was suspected that oral contraceptives were the cause that led to the development of pancreatitis. Some studies have found a relationship between pancreatitis and oral contraceptives, especially in women over 40 years of age.^[12]^ Ultrasound do not always help in diagnosing the disease, although it was the 1st investigation performed. However, studies have shown that the benefit of ultrasound is to diagnosing inflammation caused by stones or biliary dilatation. It is limited in evaluating the pancreatic tissue due to the presence of the bowel gas, as for the computed tomography it also did not have any diagnostic benefit in our case, and according to what reported computer tomography scan can detect pancreatitis with a sensitivity of 60% to 95%.^[13]^ The diagnosis was based mainly on MRCP imaging, which was able to detect the presence of congenital anomalies, MRCP is considered one of the best radiological techniques that help in diagnosing a pancreatic divisum.^[14]^ As we found, neither the clinical history nor conventional methods helped in diagnosing the condition, as in 10% to 30% of cases of pancreatitis it is difficult to know the cause of pancreatitis based on the clinical history and standard diagnostic tests.^[15]^ It is also necessary to scan the whole pancreatic areas while examination, because pancreatitis may appear in areas without the others.^[9]^

Conservative management is not always considered as an effective treatment for pancreatitis, so the principal treatment was papillotomy of the minor papilla with short-term stenting by ERCP and this management shows that it is effective and safe for associated acute recurrent pancreatitis, despite the therapeutic benefit of this intervention, but it has complications. Therefore, the patient developed postERCP pancreatitis, which is considered the most common complication related to ERCP, with an incidence of 2% to 20%.^[15]^

## 4. Conclusion

This case highlights the importance of considering pancreas divisum as one of the causes of pancreatitis, particularly in older patients, or patients who do not have a history of alcohol consumption, gallstones, or any other prevalent risk factors. It also highlights on the importance of each radiological examination separately, with particular focus on the essential radiological investigation that can identify the diagnosis accurately.

### 4.1. Take-away lessons

This case highlights the importance of considering congenital pancreatic anomalies, particularly pancreas divisum, in patients presenting with recurrent or unexplained pancreatitis. Although uncommon, early recognition through appropriate imaging and timely endoscopic management can prevent long-term morbidity. Clinicians should maintain a high index of suspicion when common etiologies of pancreatitis are excluded. Furthermore, patient-centered outcomes demonstrate that targeted interventions can significantly improve quality of life, underscoring the value of individualized diagnostic and therapeutic approaches.

### 4.2. Patient perspective

The patient reported a significant improvement in her quality of life following the endoscopic intervention. Prior to diagnosis and treatment, she experienced severe abdominal pain, recurrent diarrhea, and persistent vomiting that interfered with her daily activities and overall well-being. After undergoing ERCP with minor papilla sphincterotomy and stent placement, she described rapid relief of symptoms and the ability to resume normal nutritional intake. At follow-up, she emphasized the importance of timely recognition and treatment of her condition, as the procedure was perceived as life-changing and restored her functional capacity.

## Author contributions

**Conceptualization:** Nagham Alsaid Abdullah Altwer.

**Data curation:** Nagham Alsaid Abdullah Altwer.

**Formal analysis:** Nagham Alsaid Abdullah Altwer.

**Investigation:** Nagham Alsaid Abdullah Altwer.

**Methodology:** Nagham Alsaid Abdullah Altwer.

**Project administration:** Nagham Alsaid Abdullah Altwer.

**Resources:** Nagham Alsaid Abdullah Altwer.

**Software:** Nagham Alsaid Abdullah Altwer.

**Supervision:** Bayan Alsaid

**Validation:** Bayan Alsaid

**Visualization:** Bayan Alsaid

**Writing – original draft:** Nagham Alsaid Abdullah Altwer.

**Writing – review & editing:** Nagham Alsaid Abdullah Altwer.
